# Supplementary material for: Personal response to immune checkpoint inhibitors of patients with advanced melanoma explained by a computational model of cellular immunity, tumor growth, and drug
Source: PLoS One. 2019 Dec 26;14(12):e0226869. doi: 10.1371/journal.pone.0226869 (PMC6932803; doi:10.1371/journal.pone.0226869)
Supplement: S1 Text — Table A. Clinical Information for Patient O' Table B. Values and References for the Generic Model Parameters Fig A. Sensitivity analysis of Patient O’s model for the parameter pc Fig B. Sensitivity analysis of Patient O’s model for the parameter kC03 (DOCX) [file pone.0226869.s001.docx]

**PLoS One Supporting Information Appendix S1**

Article title: Personal response to pembrolizumab of patients with advanced melanoma, explained by a computational model of cellular immunity, tumor growth and drug

Authors: D. Perlstein, O. Shlagman, Y. Kogan, K. Halevi-Tobias, A. Yakobson, I. Lazarev, Z. Agur.

The following Supporting Information is available for this article:

S1 Table A. Clinical Information for Patient O'

S1 Table B. Values and References for the Generic Model Parameters

S1 Fig. A. Sensitivity analysis of Patient O’s model for the parameter *p_c_*

S1 Fig. B. Sensitivity analysis of Patient O’s model for the parameter $k_{C}^{03}$

S1 Text. Detailed mathematical modeling, parametrization, generation and analysis of virtual populations

**S1 Table A**. **Clinical Information for Patient O'**

|  | **Tumor size/site (mm)** | | | | |
| --- | --- | --- | --- | --- | --- |
| **Date of tumor measurement/ treatment:** | **Right frontal mediastinum** | **Near mediastinum** | **Right base hemitorex** | | **Pleural** |
| 03/06/14 | 135 x 101 |  |  | |  |
| 06/07/14 | 136 x 107 |  |  | |  |
| *Treatment: Surgery 08/07/14* | | | | | |
| 03/08/14 | 40 x 36 |  |  |  | |
| *Treatment: Radiotherapy 27/8/14 – 1/10/14* | | | | | |
| 04/11/14 | 20 x 10 | 36 x 25 | 120 x 47 |  | |
| *Treatment: Start immunotherapy 20/11/14* | | | | | |
| 21/12/14 | 20 x 9.4 | 57 x 49 | 133 x 54 | 4 | |
| 23/02/15 | 18 x 6 | 33 x 29 | 102 x 35 | 10 | |
| 04/06/15 | 15 x 5 | 38 x 36 | 98 x 41 | 11 | |
| 12/08/15 | 15 x 4 | 45 x 40 | 100 x 49 | 12 | |
| 10/12/15 | 9 | 42 x 38 | 86 x 43 | 10 | |
| 12/04/16 | 8 | 40 x 37 | 85 x 42 | 9 | |
| 01/08/16 | 8 | 36 x 34 | 83 x 40 | 8 | |
| 02/01/17 | 5 | 36 x 32 | 78 x 40 | 8 | |
| 05/07/17 | 0 | 32 X 27 | 77X 31 | 0 | |
| 01/01/18 | 0 | 32 X 27 | 76 x 30 | 0 | |

**S1 Table B. Values and References for the Generic Model Parameters**

| **Parameter** | **Description** | **Value [units]** | **Reference** |
| --- | --- | --- | --- |
| $k_{p}$ | Clearance rate of pembrolizumab from the blood | $0.0011[h^{-1}]$ | [12] |
| $V_{d}$ | Volume of distribution of pembrolizumab | $7.75 [L]$ | [12] |
| $M_{mol}$ | Pembrolizumab molecular mass | $2.47\times{10}^{-22}kg$ | [12] |
| $T_{inj}$ | Period of pembrolizumab infusion | $0.5 [h]$ | [12] |
| $C_{L}$ | Pembrolizumab clearance | $209 [mL/day]$ | [12] |
| $p_{N}$ | Cell division rate of N cells | $0.0117 [h^{-1}]$ | [7] |
| $d_{N}$ | Death rate of N cells | $3.1\times{10}^{-4} [h^{-1}]$ | [7] |
| $p_{SCM}$ | Cell division rate of SCM cells | $0.0156 [h^{-1}]$ | [7] |
| $d_{SCM}$ | Death rate of SCM cells | $4.96\times{10}^{-5} [h^{-1}]$ | [7] |
| $p_{CM}$ | Cell division rate of CM cells | $0.0112 [h^{-1}]$ | [7] |
| $d_{CM}$ | Death rate of CM cells | $4.96\times{10}^{-4} [h^{-1}]$ | [7] |
| $p_{EM}$ | Cell division rate of EM cells | $0.0062 [h^{-1}]$ | [7] |
| $d_{EM}$ | Death rate of EM cells | $0.0021 [h^{-1}]$ | [7] |
| $p_{E}$ | Cell division rate of E cells | $0.0045 [h^{-1}]$ | [7] |
| $d_{E}$ | Death rate of E cells | $0.021 [h^{-1}]$ | [7] |
| $k_{D}$ | The rate of migration into the LN and maturation of activated DCs | $0.027 [h^{-1}]$ | [13] |
| $\mu$ | The death rate of mature DCs | $0.014 [h^{-1}]$ | [13] |
| $\alpha_{l}$ | The probability for an activated DC to survive the journey to the LN and mature | $0.3 [h^{-1}]$ | [13] |
| z_0_ | ratio of cytotoxic effectivity of EM cells to that of E cells | 0.14 | [7] |


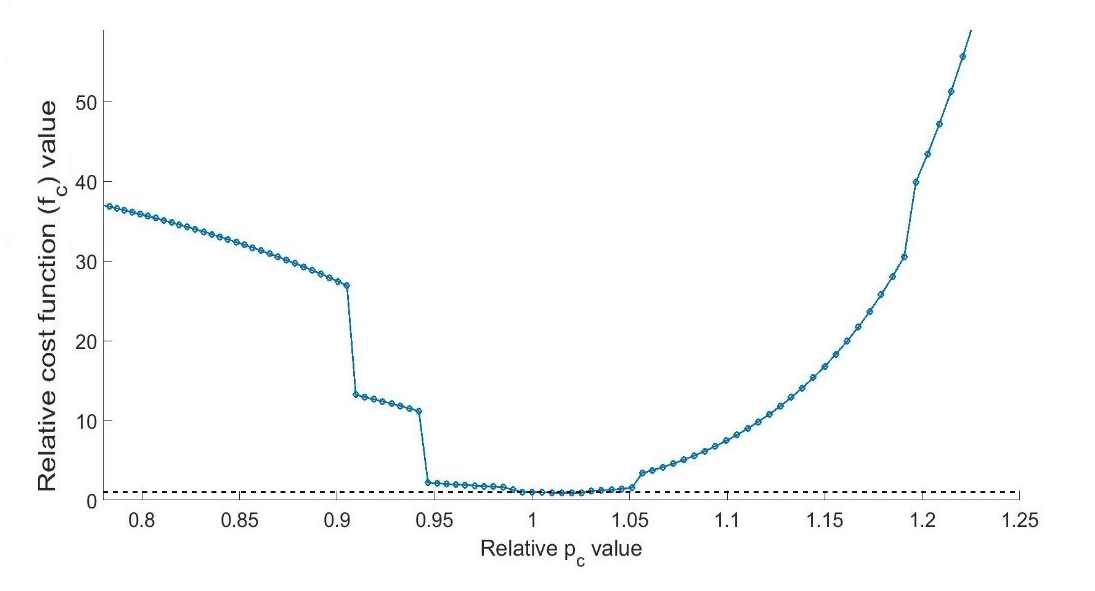


**S1 Fig. A. Cost function values relative to the value at best fit point plotted versus the value of *p_c_* relative to its corresponding value at best fit point.** The figure shows a steep increase in cost when changing the value of *p_c_* at best fit point. For reference, the black dashed line represents the value 1 of the ordinate. It can be seen that the minimum of the cost function is obtained very closely to the value 1 of the abscissa.


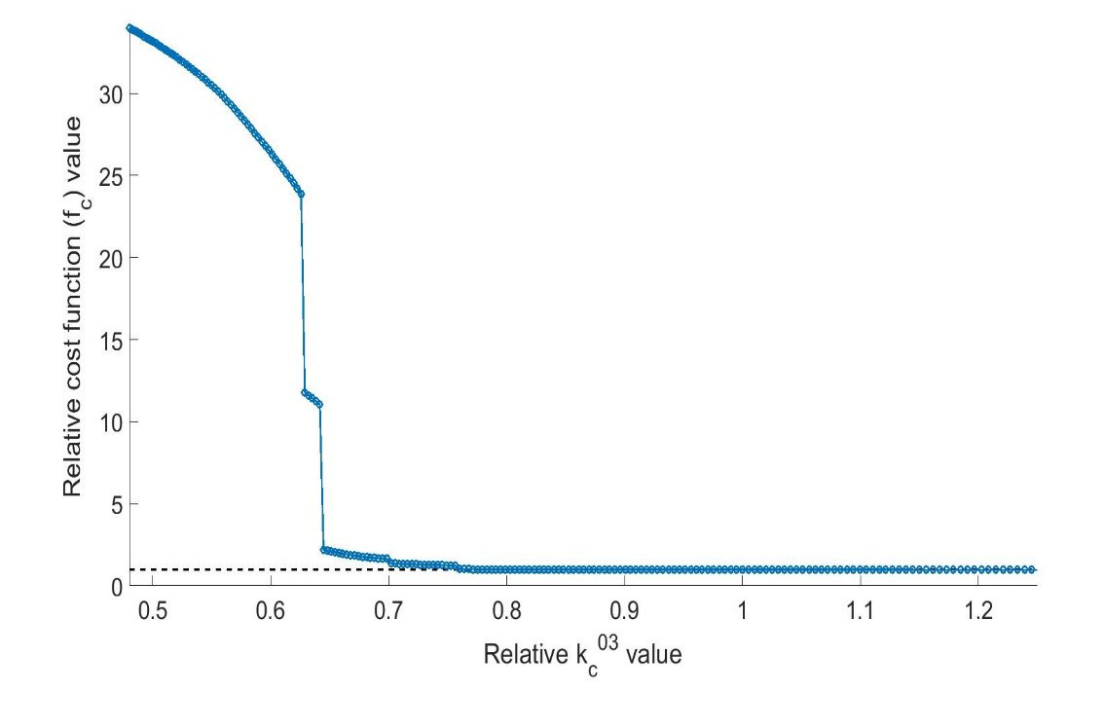


**S1 Fig. B. Cost function value relative to the value at best fit point, as a function of the value of *k_c_^03^* relative to its respective value at best fit point.** The figure shows the steep increase in relative cost function values when decreasing ***k_c_^03^*** below its value best fit point, while increasing of ***k_c_^03^*** above the value at best fit point maintains the cost unchanged, due to the system reaching saturation.

**S1 Text.** Detailed mathematical modeling, parametrization, generation and analysis of virtual populations

**Full mathematical description of the model**

We formalized the dynamics of the cellular immune system as a system of

$5\cdot m-6$ Ordinary Differential Equations (ODEs), where $m=25$is the maximal number of divisions for a single cell. Two equations describe dynamics of the DC, one equation describes the cancer growth and one equation describes the PK of pembrolizumab. The remaining $5\cdot m-10$ ODEs describe the dynamics of the T cell developmental pipeline, extending over five compartments that represent different developmental stages: Stem Cell Memory (SCM), Central Memory (CM), Effector Memory (EM), Effector (E) and Exhausted (EXH), enumerated by $i=1,\ldots,5$, respectively, whereas $i=0$ refers to the naïve antigen-specific compartment. For simplicity, the effect of partly exhausted cells is represented by the effect of pembrolizumab on the weakening of the inhibitory potency of PD-L1/PD-1 binding on EM and E cells. Fully exhausted cells (EXH) appear in the model equations only for reference and analysis purposes.

The ODE system for the immunity-tumor dynamics can be compactly written, as detailed below. The dynamics of DCs are represented by equations (1) and (2):

$\dot{D}_{act}=\frac{\rho D_{act}C}{D_{act}+C}-k_{D}D_{act}$ (1)

$\dot{D}_{m}=a_{l} k_{D} D_{act} -\mu D_{m}$ (2)

$D_{act}$ stands for activated DCs in the tumor site and $C$ is the number of cancer cells. The first term in equation (1) corresponds to activation due to binding of DCs to cancer cells and absorption of cancer antigens, with parameter $\rho$ depicting the immunogenicity, i.e., the basic activation rate of DCs by cancer cells. The second term in equation (1) is the migration of activated DCs from the tumor site and their maturation, $k_{D}$ being the rate at which DCs migrate from the tumor and mature. Equation (2) describes the dynamics of mature DCs ($D_{m}$) in the nearest Sentinel Lymph Node (SLN). The first term represents migration rates of $D_{act}$ into the SLN through coefficient$k_{D}$ defined in equation (1), and the probability $a_{l}$ of an activated DC to arrive successfully at the lymph node. The death rate of mature DCs is$\mu$.

The stimulation of T cells due to encounters with antigen-presenting DCs is described by the Michaelis-Menten-type function, $f_{i}$, where $i$ ranges over the T lymphocyte differentiation compartments.

$f_{i}=\left\{ \begin{matrix} i=1,2; & \frac{D_{m}}{D_{m}+T_{LN}} \\ i=3,4,5; & 1 \end{matrix} \right\}, T_{LN}\equiv\sum_{j} \left( T_{SCM,j}+T_{CM,j} \right)$. (3)

Compliant with our assumptions (as listed in the Materials and Methods section), the function $f_{i}$ describes the interactions of DCs with T cells, for $i=1,2$ (compartments SCM and CM) only. $T_{LN}$ is the total number of antigen-specific T cells in the SLN. The variables $T_{SCM,j}\mathrm{and} T_{CM,j}$ respectively stand for the number of SCM and CM cells that completed *j* divisions. For simplicity, we assume that the number of naïve cells, $N,$ remains constant throughout the immune response.

Equation (4) is the formalization of the general T cell dynamics for $i=1,\ldots,5$. It describes the changes of the number of CD8^+^ T cells in the sub-compartment$T_{i,j}$, denoting the number of T cells in the $i$^th^ differentiation compartment that have undergone $j$ divisions:

$\dot{T}_{i,j}=2a_{i,j-1} p_{i,j-1} T_{i,j-1} f_{i}+2\left( 1-a_{i-1,j-1} \right) p_{i-1,j-1}f_{i-1} T_{i-1,j-1}-$ (4)

$-\left( p_{i,j} f_{i}+d_{i,j} \right) T_{i,j}-k_{C}^{0i}\Gamma_{i,j}^{C}$, where $k_{C}^{0i}=0$ for *i≠(3,4)* .

The first term on the right-hand side (r.h.s) of equation (4) describes the increase in the number of T cells due to self-renewal at this state, where $a_{i,j}$ denote the self-renewal probability, i.e., the probability of generating two daughter cells without differentiating, and $p_{i,j}$ denotes the basic rate of cell division. This first term depends on$T_{i,j-1}$, the number of T cells in the same compartment $i$ and at the previous generation,$j-1$. The coefficient 2 in equation (4) corresponds to two daughter cells generated in each division. The cell division rate $p_{i,j}$ decreases linearly with cell divisions, due to senescence, e.g., by telomere shortening. For the compartments SCM and CM we define:

$p_{1,j}=p_{1}^{0}(1-\frac{j}{m}), p_{2,j}=p_{2}^{0}(1-\frac{j}{m})$ . (4.1)

However, in the PD-1 expressing cells, namely, the EM and E compartments$(i=3,4$), in addition to the reduction due to senescence, the cell division rates are also reduced by PD-1/PD-L1 binding.

Hence, we define:

$p_{3,j}=p_{3,j}^{0}-k_{C}^{03}\Omega_{C},$ (4.2)

$p_{4,j}=p_{4,j}^{0}-k_{C}^{04}\Omega_{C},$ (4.3)

Here $p_{i,j}^{0}$ is the cell division rate of the T cell population $i,j$ in the absence of inhibition by cancer cells:

$p_{i,j}^{0}=p_{i}^{0}(1-\frac{j}{m})$, (4.4)

describing reduction in divisive capacity due to senescence alone.

The second term in equations (4.2) and (4.3) describes the decrease in cell division of EM and E cells due to inhibition by cancer cells. In this term, the variable

$\Omega_{C}\equiv\frac{C}{C+\sum_{j} \left( T_{3,j}+T_{4,j} \right)}$ (4.5)

evaluates the overall portion of inhibited EM and E cells, those whose PD-1 receptors are bound by PD-L1 ligands on tumor cells.

The parameters $k_{C}^{0i}$ *(i=3,4)* define the binding rates of cancer to EM and E cells, respectively, de facto representing the inhibitive potency of the cancer cells. This parameter is included in all three effects of cancer cells, namely, reduction in cell division and cytotoxicity of EM and E cells (described in detail below), and induction of apoptosis in these cells. The second term on the right hand side of equation (4) describes differentiation from the previous T cell compartment ($i-1$), where $(1-a_{i-1,j-1})$ is the probability of differentiation of a $T_{i-1,j-1}$ cells into a $T_{i,j}$ cell. The coefficient 2 again denotes two daughter cells generated in each division. The third term on the right hand side of equation (4) describes reduction in the number of $T_{i,j}$ cells due to natural death ($d_{i,j}$ being the death rate) and cell divisions (the cell division rate, $p_{i,j}$ multiplied by the stimulation function $f_{i}$defined in equation (3).

The last term in equation (4) stands for the direct inhibition of T cells by cancer cells, i.e. apoptosis of $T_{i,j}$ cells due to binding to cancer cells; $\Gamma_{i,j}^{C}$ represents the overall extent of inhibition due to binding of cancer cells to $T_{i,j}$ cells, using a Michaelis-Menten term:

$\Gamma_{i,j}^{C}=\left\{ \begin{matrix} i=3,4; & \frac{T_{i,j}C}{C+\sum_{j} \left( T_{3,j}+T_{4,j} \right)} \\ i=1,2,5; & 0 \end{matrix} \right\}$ (4.6)

The fully exhausted cells $T_{EXH}(i=5)$ do not replicate and do not have any effector functionalities, nor are they affected by PD-L1. Therefore in equation (4) we define $p_{5,j}=0$. As a result, the first and the last terms in equation (4) vanish for these cells, and this equation simplifies to:

$\dot{T}_{5,j}=2\left( 1-a_{4,j-1} \right)p_{4,j-1}T_{4,j-1}-d_{5,j}T_{5,j}$ (4.7)

The dynamics of the tumor load, $C,$ are described by the following equation:

$\dot{C}=p_{C}C^{\alpha_{C}}-\frac{\sum_{j} \left( k_{3}^{E}T_{3,j}+k_{4}^{E}T_{4,j} \right)C}{C+\sum_{j} \left( T_{3,j}+T_{4,j} \right)} .$ (5)

The first term on the right hand side of equation (5) depicts the spontaneous tumor growth of a power-law type, where $\alpha_{C}$ is the governing exponent ($\alpha_{C}=\frac{2}{3}$ in current implementation), and $p_{C}$ is the rate of spontaneous growth. The second term in equation (5) describes inhibition of the tumor by the immune system, where $k_{3}^{E} \mathrm{and} k_{4}^{E} \mathrm{are}$the coefficients of cancer cell kill by EM and E cells, respectively. These coefficients are functions of the binding rates by cancer cells to PD-1 receptors, $k_{C}^{0i} (i=3,4)$:

$k_{3}^{E}=z_{0}k_{0}^{E}-k_{C}^{03}\Omega_{3}^{C},$ (5.1)

$k_{4}^{E}=k_{0}^{E}-k_{C}^{03}\Omega_{4}^{C}.$ (5.2)

The parameter $k_{0}^{E}$ in equations (5.1) and (5.2) is the basic killing rate (representing maximal cytotoxicity) of the effector cells, while $z_{0}$ is the ratio of effectivity of EM cells to that of E cells. The negative terms in equations (5.1) and (5.2) reflect the decrease in cytotoxicity of both E and EM cells due to binding of PD-L1+ tumor cells to PD-1 expressing T cells. Parameters $\Omega_{3}^{C}\mathrm{and}\Omega_{4}^{C}$ are similar to $\Omega_{C}$, but defined per single compartment:

$\Omega_{3}^{C}\equiv\frac{C}{C+\sum_{j} T_{3,j}}, \Omega_{4}^{C}\equiv\frac{C}{C+\sum_{j} T_{4,j}}$ . (5.3)

Denoting by $P$ the concentration of free pembrolizumab in blood, we describe the PK of pembrolizumab by the one-compartment linear model, as follows**:**

$\dot{P}={\sum_{n} r}_{p}\cdot\delta\left( t-3 \cdot n\cdot weeks \right)-k_{p}\cdot P.$ (6)

Here, $r_{p}$is the dose of applied pembrolizumab, assumed to be piecewise constant (equal to zero between the infusions). The second term represents first-order clearance of pembrolizumab, where $k_{p}$ is the rate of elimination.

To account for the effect of pembrolizumab on the reinvigoration of exhausted T cells in a simplified way, we modify the inhibition terms $\Gamma_{3,j}^{C}, \Gamma_{4,j}^{C}$ and $\Omega_{C}$ in equations (4.3) and (4.4), as described below. First, we take account of the number of cells which carry a sufficiently large number of blocked (by pembrolizumab antibodies) PD-1 receptors, preventing cell blockage by PD-L1. These blocked EM cells are denoted$T_{b3,j}$ and the blocked E cells are denoted $T_{b4,j}$. The blocked cells are computed by:

$T_{b3,j}\equiv\frac{PT_{3,j}}{P+\sum_{j} \left( T_{3,j}+T_{4,j} \right)}; {\begin{aligned} \\ T \end{aligned}}_{b4,j}\equiv\frac{PT_{4,j}}{P+\sum_{j} \left( T_{3,j}+T_{4,j} \right)}$. (7)

Subsequently, we obtain the modified forms of the inhibition terms:

$\Gamma_{3,j}^{C}=\frac{\left( T_{3,j}-T_{b3,j} \right)C}{C+\sum_{j} (T_{3,j}+T_{4,j}-T_{b3,j}-T_{b4,j})},$ (8)

$\Gamma_{4,j}^{C}=\frac{\left( T_{4,j}-T_{b4,j} \right)C}{C+\sum_{j} (T_{3,j}+T_{4,j}-T_{b3,j}-T_{b4,j})}$, (9)

$\Omega_{C}=\frac{C}{\frac{C}{\left[ 1-\frac{P}{P+\sum_{j} \left( T_{3,j}+T_{4,j} \right)} \right]}+\sum_{j} (+T_{3,j}+T_{4,j})}.$ (10)

The modifications of $\Gamma_{3,j}^{C}, \Gamma_{4,j}^{C}$ and $\Omega_{C}$, shown in equations (8)-(10) were obtained by replacing $T_{3,j}$ and $T_{4,j}$ from equations (4.3) and (4.4) in the Materials and Methods section by $(T_{3,j}-T_{b3,j})$ and $(T_{4,j}-T_{b4,j})$, respectively. This modification stands for the weakening, by the drug, of the inhibitory potency of PD-L1/PD-1 binding. Equation (10) describes the effect of pembrolizumab in reinvigoration of partly exhausted effector cells, effectively being realized in the loss of division capacity in EM and E cells. Thus, by modifying$\Gamma_{3,j}^{C},\Gamma_{4,j}^{C}$ and $\Omega_{C}$, then substituting the modified terms into equations (4)-(5.2), the new definitions for the inhibition terms account for all the restoring effects of pembrolizumab. These modifications complete the combined model for the tumor-immune system interactions and the effects of pembrolizumab on the reduction of the direct inhibition and the loss of effector functions.

**Estimation of general parameters of the immune system**

To evaluate the division rates of immune cells we used the data extracted from Fig. 5c in Geginat et al., 2003 [7]. In these experiments, CD8+ T cells of the T cells subsets SCM, CM, EM and E were cultured *ex vivo*, with antigen presenting DCs, and Bromodeoxyuridine (BrdU) was used to measure the number of dividing CD8+ T cells and the number of cells dying during a week of stimulation. We estimated the cell division and death rates by fitting a simple mathematical model describing the essential dynamics of T cells division and death, as observed in this experiment. The ODE describing, for example, the SCM cells dynamics in this experiment were taken to be:

$\dot{T}_{SCM}=(p_{SCM}-d_{SCM} )T_{SCM}$, (11)

where $T_{SCM}$ is the number of $T_{SCM}$ cells at time t, $p_{SCM}$ is the division rate of $T_{SCM}$ cells, and $d_{SCM}$ is the death rate of $T_{SCM}$ cells. The solution to equation (10) is an exponential growth function of $T_{SCM}$ cells:

$T_{SCM}\left( t \right){=T}_{0}e^{\left( p_{SCM}-d_{SCM} \right)t}$ (12)

Here, $T_{0}$ stands for the initial number of $T_{SCM}$ cells. Taking the associated values from the data in Fig. 5c in [7], and substituting the measured initial $(t=$0) and final ($t=168h$) values of $T_{SCM}$ cells into expression (12), an estimate for $p_{SCM}{-d}_{SCM}$ can be obtained. Using the same data [7] to evaluate the percentage of dead cells after a week, one obtains:

$p_{SCM}=0.0156h^{-1},d_{SCM}=4.96\times{10}^{-5} h^{-1}$ .

Applying a similar calculation to the other T cell compartments, based on equation (12), we derived the death and cell division rates of central memory cells, effector memory cells and effector cells:

$p_{CM}=0.0112h^{-1},d_{CM}=4.96\times{10}^{-4}$,

$p_{EM}=0.0062h^{-1},d_{EM}=0.0021h^{-1}$,

$p_{E}=0.0045h^{-1},d_{EFF}=0.021h^{-1}$.

The parameters related to dendritic cells dynamics which were obtained from the literature are: $k_{D}$ - the rate of migration into the lymph nodes and maturation of activated DCs, $a_{l}$ - the probability for an activated dendritic cell to survive the journey to the lymph node and mature, and $\mu$ - the death rate of mature dendritic cells. These parameters were evaluated for migration of injected dendritic cells from the dermis to the tumor sentinel lymph node, as described in [8].

$k_{D}=0.027h^{-1},a_{l}=0.3,\mu=0.014h^{-1}$.

**General PK parameters of pembrolizumab**

We used the Food and Drug Administration (FDA) report on pembrolizumab to obtain the values for the PK parameters of our model [9]. In this FDA report, the clearance coefficient of pembrolizumab ($C_{L}$) is evaluated as $209 mL/day$, the volume of distribution ($V_{d}$ ) as $7.75L$, and the molecular weight as 149kDa. The clearance rate of pembrolizumab, $k_{p}$ in (e.g., in equation (6)), is obtained via division of the clearance coefficient, $C_{L}$, by the volume of distribution $V_{d}$, which results in $k_{p}=0.0011 h^{-1}$. Transforming the molecular weight of the injected pembrolizumab dose into kilograms we obtain the molar mass of pembrolizumab$M_{mol}=2.47\times{10}^{-22} kg$. Denoting the dosage (in mg) by$D$, and the mass of the patient by $m$, we derive a formula for the number of pembrolizumab molecules for a single injection, $p_{d}=Dm/(V_{d}M_{mol})$.

**Estimation of patient-specific parameters**

As described in the Materials and Methods section, values for most parameters in our model were initially estimated, based on information in the literature, and assumed to be general for all patients. Other parameters were allowed to assume, for each patient, values from relatively wide respective ranges. The parameters related to the immune system, whose values were estimated from the literature, comprise the following groups: (i) the basic cell division rates of functional CD8^+^ T cells - $p_{SCM}, p_{CM}, p_{EM},p_{E}$; (ii) the death rates of functional CD8^+^ T cells - $d_{SCM},d_{CM},d_{EM},d_{E}, d_{EX}$; (iii) parameters associated with the dendritic cells: the probability of activated dendritic cells to mature- α_l_, the migration rate of dendritic cells to the lymph node - *k_D_*, and the death rate of dendritic cells - µ; (iv) PK parameters: clearance rate of pembrolizumab from the body - k_P_, and pembrolizumab dosage constant – *r_P_*; and (v) miscellaneous parameters: a constant number of naïve cells – N, and z_0_ - the ratio of effectivity of EM cells to that of E cells. Initially, nine parameters were deemed specific: the killing rate of tumor cells by CD8+ T cells, $k_{E}^{0}$ [2]; the basic inhibition rates of immune cells by tumor cells, $k_{C}^{0i}$ *(i=3,4)* [3]$;$cancer immunogenicity (i.e., activation rate of DCs by tumor antigens), $\rho$ [4]; the tumor growth rate coefficient, $p_{c}$ [5]; and the parameters $a_{i}$ *(i=1,..,4)*, representing the probability of self-renewal in the compartments SCM, CM, EM and E, respectively (but note that in order to retrieve results in [6], we also varied among patients the E replication rate, $p_{E}$). Values for $a_{i}$ were not obtainable from current literature. They were thus estimated as those that best fit the tumor load data of patient O', who nevertheless, depicts an uncharacteristic tumor load dynamics, so the best fit obtained for that patient yielded values that are possibly uncharacteristic for these parameters (see Discussion section). While biologic intuition suggests high likelihood for the other specific parameters to indeed be such, it is unclear as yet whether the parameters $a_{i}$ actually vary among the patient population. It is a main scope of a work in progress to shed further light on the values that these parameters can receive.

**Algorithm for searching global minima of a cost function**

The operation stages of the tailored algorithm are the following.

For each parameter whose value can be estimated based on the literature, define a corresponding narrow range that averages about the estimated value.

For each of the other parameters, define some average value by combining biologically realistic assumptions (for example, a plausible rate of spontaneous cancer growth, etc.) with results of preliminary simulations, yielding parameter ranges over which the system shows varying dynamic behavior. Then, define a relatively wide range (e.g., max_value/min_value=103) about the average values.

Begin the first search by assigning each parameter its respective average value.

The search is an iterative step process, where each step includes transition to a new point in parameter space, evaluation of the cost function in that new point, and a decision regarding the following step.

In each iterative step:

If the previous step decreased the defined cost function by more than a first threshold, then from the current point in parameter space perform a step of the same size and in the same direction as the previous one, and calculate the new cost function.

If the previous step increased the cost function value by more than a second threshold, then from the previous point in parameter space perform a step of the same size and in a direction opposite to that attempted in the latest step, and calculate the new cost function.

If none of the above occur, do the following:

Randomly choose the number and identity of parameters to which apply a change in value.

For each selected parameter, randomly choose a multiplicative factor to multiply the current value of the parameter by. The average step size depends on the chosen distribution, the range size of the parameter, the number of iterations performed (via a ‘temperature’ that cools over time), and on meeting threshold values associated with the cost function, e.g., decreasing the steps with decreasing cost function values.

If the new value of any parameter is out of its range, choose instead a value within range, close to the relevant range edge.

The cost function calculates the root mean square error between data and simulation, and combines it with positive and negative costs associated with the graph shape, proximity to the average values of the parameters, and possibly other criteria.

Keep a record a number of parameter-space points that currently yield the lowest cost function values.

The search is repeated multiple times, with different starting points, for example, points that evenly cover parameter space, points that are linear combinations of lowest cost function points of previous searches, etc.

The above-described tailored algorithm, was implemented on data of other patients as well (work in progress), and reached marginally better results (a margin of 0.5-1 percent) than any built-in matlab algorithm attempted as comparison. In some cases the same results were obtained, but the tailored algorithm converged more quickly to the associated point of low cost function value.

**Sensitivity of Patient O’s model parameters**

Following the completion of Patient O’s model, a sensitivity analysis was performed, in order to verify that the parameter estimation for this patient’s model was the most plausible one, and that the estimated parameters are not redundant or non-identifiable. A large number of simulations was performed to check for possible un-smooth changes in the time course of the simulated cancer dynamics with the change of any of the parameters. No such un-smoothness was found. As the cost function itself was defined so as to not explicitly introduce any un-smoothness in the model parameters, our basic sensitivity analysis was performed by numerically examining the partial derivatives of the cost function, and deducing conclusions therefrom regarding all possible directions, as is warranted for a smooth function.

To this end, we varied the values of five specific parameters between 0.2 and 5 fold their value at the best-fit point. Each parameter was sampled uniformly in its respective range, while keeping all other model parameters constant. We calculated the cost function (see Materials and Methods) over these ranges. For four of the five studied parameters, a clear minimum in cost function was obtained at the best fit point, where the cost function increases by 1.5 to >20 fold for changes of up to 50% in the parameter value (see Materials and Methods). This result shows that these evaluated four parameters are the most probable for Patient O’s model within the studied range. For one parameter, $k_{C}^{03}$, the value evaluated for Patient O’ was a one-sided minimum. That is to say that decreasing the value of $k_{C}^{03}$ increased the cost considerably, while increasing its value caused no change in the cost, apparently, due to the system reaching saturation at those values. Thus, the estimated patient-specific $k_{C}^{03}$ value is the minimal among its plausible values. This analysis, combined with the exhaustive coverage of the biologically feasible parameter ranges, implemented by our search algorithm, points towards the existence of a single global optimum for the parameter values of patient O’. Figs. S1 A and S1 B show examples of the computational analysis of the accuracy of model fit to the clinically evaluated longitudinal tumor size measurements of Patient O’, when the parameters *p_c_* and *k_c_^03^* are varied in the respective range as described above. Further analysis will clarify the identifiability of the values of specific parameters for a single patient (in preparation).

**Virtual populations modelling**

**Selecting the members for the virtual populations**

According to the scheme for creating the virtual populations, each potential member in each of the virtual populations was initially assigned a set of values for his/her specific parameters, and a random baseline tumor load, which is the tumor size at the time of first pembrolizumab administration. The baseline tumor load was defined as the Sum of Longest Diameters (SLD) of the potential member's tumor lesions, and was randomly chosen from a tailored distribution designed to yield tumors having similar sizes to those of the cohort depicted in [6]. Each potential member was then assigned a random integer number between 1 and 4 to represent the total number of his/her tumor lesions; one of the lesions was assumed to be substantially larger than the other(s). We then performed a preliminary simulation run for each patient, taking the initial tumor load at simulation onset to be smaller than his/her assigned baseline tumor load, by a factor of about 2/3. This run simulated 32 weeks during which pembrolizumab was not administered. As the system characteristically reached a "natural" state in less than two simulated weeks, it was checked whether the tumor size at onset attained the designated baseline tumor load at some point during the subsequent 30 weeks of simulation. If it did, then that member was selected for the reference virtual population. A simulation run was then performed for each selected member, where assigned initial conditions corresponded to the conditions obtained in the preliminary simulation for that member, at the time of reaching his/her baseline tumor load.

**Classifications of patients in virtual populations according to their response and determining the classification accuracy**

The ratio between the number of responders and non-responders in each virtual population varies, based on the identity and distribution of the parameters chosen to be patient-specific for that simulation. As this can introduce various artifacts in the classification scheme, a weighing was performed according to the ratio between the sizes of the two groups. Accordingly, if there are, for example, three times as many responders as non-responders in a simulated population, each non-responder will weigh (or count) thrice in that simulation.

In accordance with [10, 11], the points representing the patients were scattered on the reinvigoration to baseline-load plane, and tagged based on their respective responses. All lines that pass through the origin (to be consistent with the methodology and hypothesis in [11]) and separate the points into two non-empty groups were drawn, and the accuracy of the classification obtained by each line was determined by calculating the (weighted) portion of correctly classified patients, i.e., the portion of patients whose model-simulated response corresponded to their classified response. In agreement with the methodology in [11], the line that most accurately classified the patients was chosen, and the associated portion of correctly classified patients defined the classification accuracy.

REFERENCES

1. Agur Z, Halevi-Tobias K, Kogan Y, Shlagman O. Employing dynamical computational models for personalizing cancer immunotherapy. Expert Opin Biol Th. 2016;16(11):1373-85. doi: 10.1080/14712598.2016.1223622. PubMed PMID: WOS:000385562100007.

2. Kaczorowski KJ, Shekhar K, Nkulikiyimfura D, Dekker CL, Maecker H, Davis MM, et al. Continuous immunotypes describe human immune variation and predict diverse responses. Proc Natl Acad Sci U S A. 2017;114(30):E6097-E106. doi: 10.1073/pnas.1705065114. PubMed PMID: 28696306; PubMed Central PMCID: PMCPMC5544312.

3. Igawa S, Sato Y, Ryuge S, Ichinoe M, Katono K, Hiyoshi Y, et al. Impact of PD-L1 Expression in Patients with Surgically Resected Non-Small-Cell Lung Cancer. Oncology. 2017;92(5):283-90. doi: 10.1159/000458412. PubMed PMID: 28222447.

4. Blankenstein T, Coulie PG, Gilboa E, Jaffee EM. The determinants of tumour immunogenicity. Nature Reviews Cancer. 2012;12:307. doi: 10.1038/nrc3246.

5. Todosi A, M., HuÈ›anu I, Gavrilescu M, M., Moscalu M, Ferariu D, Scripcariu V. Assessment of Tumor Parameters as Factors of Aggressiveness in Colon Cancer. Journal of Surgery [Jurnalul de Chirurgie]. 1970;10:1-5.

6. Huang AC, Postow MA, Orlowski RJ, Mick R, Bengsch B, Manne S, et al. T-cell invigoration to tumour burden ratio associated with anti-PD-1 response. Nature. 2017;545(7652):60-5. doi: 10.1038/nature22079. PubMed PMID: 28397821; PubMed Central PMCID: PMCPMC5554367.

7. Geginat J, Lanzavecchia A, Sallusto F. Proliferation and differentiation potential of human CD8+ memory T-cell subsets in response to antigen or homeostatic cytokines. Blood. 2003;101(11):4260-6. doi: 10.1182/blood-2002-11-3577. PubMed PMID: 12576317.

8. Seidel JA, Otsuka A, Kabashima K. Anti-PD-1 and Anti-CTLA-4 Therapies in Cancer: Mechanisms of Action, Efficacy, and Limitations. Front Oncol. 2018;8:86. doi: 10.3389/fonc.2018.00086. PubMed PMID: 29644214; PubMed Central PMCID: PMCPMC5883082.

9. Kang SP, Gergich K, Lubiniecki GM, de Alwis DP, Chen C, Tice MAB, et al. Pembrolizumab KEYNOTE-001: an adaptive study leading to accelerated approval for two indications and a companion diagnostic. Annals of Oncology. 2017;28(6):1388-98. doi: 10.1093/annonc/mdx076.

10. Michot JM, Bigenwald C, Champiat S, Collins M, Carbonnel F, Postel-Vinay S. Immune-related adverse events with immune checkpoint blockade: a comprehensive review. Eur J Cancer. 2016;54. doi: 10.1016/j.ejca.2015.11.016.

11. Shvartser-Beryozkin Y, Yakobson A, Benharroch D, Saute M, Feinmesser M. Malignant Melanoma in Association With a Thymic Nevus in a Patient With a Giant Congenital Nevus. Am J Dermatopathol. 2017;39(7):538-41. doi: 10.1097/DAD.0000000000000817. PubMed PMID: 28033154.

12. FDA. KEYTRUDA® (pembrolizumab) Highlights Of Prescribing Information 2017. 1-34]. Available from: <https://www.accessdata.fda.gov/drugsatfda_docs/label/2017/125514s013lbl.pdf>.

13. Kronik N, Kogan Y, Elishmereni M, Halevi-Tobias K, Vuk-Pavlovic S, Agur Z. Predicting outcomes of prostate cancer immunotherapy by personalized mathematical models. Plos One. 2010;5(12):e15482. doi: 10.1371/journal.pone.0015482. PubMed PMID: 21151630; PubMed Central PMCID: PMCPMC2999571.
