# Supplementary material for: Personal response to immune checkpoint inhibitors of patients with advanced melanoma explained by a computational model of cellular immunity, tumor growth, and drug
Source: PLoS One. 2019 Dec 26;14(12):e0226869. doi: 10.1371/journal.pone.0226869 (PMC6932803; doi:10.1371/journal.pone.0226869)
Supplement: Attachment — Submitted filename: revision_PlosOne_October2019_v01.pdf [file pone.0226869.s002.pdf]

Review for "PLOS ONE", manuscript ID: PONE-D-19-25008

Minor comments

- Regarding the parameters' estimation, authors used a simulation based method, that is the estimated parameters are those that minimise the distance between the observed tumor load and the one obtained from the simulations. The minimisation has been carried–out through an "algorithm that combines various scheme and search methods...". It would be worthwhile if authors could give more details regarding how do they implemented such algorithm together with a quantitative measure regarding the efficiency gained compared to the built–in matlab algorithms.
- Regarding the sensitivity analysis, the authors varied the parameters one by one in a given range and looked at the behaviour of the cost function. This approach does not take into account the multidimensional nature of the cost function, therefore the authors should evaluate the cost function over a multidimensional grid in which all the parameters vary simultaneously.
- Generally, point estimation provides poor information because it could vary with the simulations. For this reason, authors should provide estimated confidence intervals instead of just the single point estimation. Confidence intervals would give information regarding the robustness of the parameters' estimate together with an hint regarding the range in which the parameters should vary.
- It is not clear why the authors choose to vary the parameters in 1.63–fold range above and below the estimated baseline values, maybe this choice should be discussed a bit.
- Regarding the classification between responders and non-responders obtained in the 4 virtual populations, the plots in figure 4 do not show an evident clustering of the two groups but Table 1 gives a description of the obtained results. However, the method adopted to evaluate the classification accuracy is not given, therefore it would be worthwhile to add more details.

Typos, etc.

- Line 110: the word "among" should be used instead of "between";
- Figure 1a: $\mathrm{T_{EFF}}$ should be used instead of $\mathrm{T_E}$ because in the text the first notation has always been used. Coherently, also the notations $\mathrm{K_E}$ and δ_E should become $\mathrm{K_{EFF}}$ and $\delta_\mathrm{EFF}$as is used in the text;

- Line 193: "...with increasing differentiation index";
- Line 198: "[" should be removed;
- Line 262: the second "]" should be removed;
- Line 262: "divided into complete response (CR, not achieved...)", extended name should be added for clearness purpose, even if already defined in the introduction;
- Figure 3b: the notation in the legend should be coherent with the one in the text, thus ρ_D should be used instead of ρ;
- Line 399: the second "." should be removed;
- Table 1, line VP3, third column: at point 2. "S" should be removed.
