# Supplementary material for: Personal response to immune checkpoint inhibitors of patients with advanced melanoma explained by a computational model of cellular immunity, tumor growth, and drug
Source: PLoS One. 2019 Dec 26;14(12):e0226869. doi: 10.1371/journal.pone.0226869 (PMC6932803; doi:10.1371/journal.pone.0226869)
Supplement: Attachment — Submitted filename: Reply to Reviewers 11_11.docx [file pone.0226869.s003.docx]

PONE-D-19-25008
Personal response to immune checkpoint inhibitors of patients with advanced melanoma explained by a computational model of cellular immunity, tumor growth, and drug
PLOS ONE

**Review Comments to the Author**

1.1. Reviewer 1

Regarding the parameters' estimation, authors used a simulation based method that is the estimated parameters are those that minimise the distance between the observed tumor load and the one obtained from the simulations. The minimisation has been carried-out through an \algorithm that combines various scheme and search methods...". It would be worthwhile if authors could give more details regarding how do they implemented such algorithm together
with a quantitative measure regarding the efficiency gained compared to the built-in matlab algorithms.

Answer:

We have added a detailed specification of the tailored algorithm in the Supporting Information appendix, as follows:

The operation stages of the tailored algorithm are the following.

- For each parameter whose value can be estimated based on the literature, define a corresponding narrow range that averages about the estimated value.
- For each of the other parameters, define some average value by combining biologically realistic assumptions (for example, a plausible rate of spontaneous cancer growth, etc.) with results of preliminary simulations, yielding parameter ranges over which the system shows varying dynamic behavior. Then, define a relatively wide range (e.g., *max_value*/*min_value=10^3^*) about the average values.
- Begin the first search by assigning each parameter its respective average value.
- The search is an iterative step process, where each step includes transition to a new point in parameter space, evaluation of the cost function in that new point, and a decision regarding the following step.

In each iterative step:

- If the previous step decreased the defined cost function by more than a first threshold, then from the current point in parameter space perform a step of the same size and in the same direction as the previous one, and calculate the new cost function.
- If the previous step increased the cost function value by more than a second threshold, then from the previous point in parameter space perform a step of the same size and in a direction opposite to that attempted in the latest step, and calculate the new cost function.
- If none of the above occur, do the following:
- Randomly choose the number and identity of parameters to which apply a change in value.
- For each selected parameter, randomly choose a multiplicative factor to multiply the current value of the parameter by. The average step size depends on the chosen distribution, the range size of the parameter, the number of iterations performed (via a ‘temperature’ that cools over time), and on meeting threshold values associated with the cost function, e.g., decreasing the steps with decreasing cost function values.
- If the new value of any parameter is out of its range, choose instead a value within range, close to the relevant range edge.
- The cost function calculates the root mean square error between data and simulation, and combines it with positive and negative costs associated with the graph shape, proximity to the average values of the parameters, and possibly other criteria.
- Keep a record a number of parameter-space points that currently yield the lowest cost function values.

The search is repeated multiple times, with different starting points, for example, points that evenly cover parameter space, points that are linear combinations of lowest cost function points of previous searches, etc.

The above-described tailored algorithm, was implemented on data of other patients as well (work in progress), and reached marginally better results (a margin of 0.5-1 percent) than any built-in matlab algorithm attempted as comparison. In some cases the same results were obtained, but the tailored algorithm converged more quickly to the associated point of low cost function value.

1.2. Reviewer 1

Regarding the sensitivity analysis, the authors varied the parameters one by one in a given range and looked at the behavior of the cost function. This approach does not take into account the multidimensional nature of the cost function, therefore the authors should evaluate the cost function over a multidimensional grid in which all the parameters vary simultaneously.

Answer:

For a smooth multivariable function, the values of its partial derivatives provide sufficient information for determining its local change in any direction. Accordingly, the text below was inserted in the Supporting Information, in the “Sensitivity of Patient O’s model parameters” section, at the end of the first paragraph in the revised manuscript.

A large number of simulations was performed to check for possible un-smooth changes in the time course of the simulated cancer dynamics with the change of any of the parameters. No such un-smoothness was found. As the cost function itself was defined so as to not explicitly introduce any un-smoothness in the model parameters, our basic sensitivity analysis was performed by numerically examining the partial derivatives of the cost function, and deducing conclusions therefrom regarding all possible directions, as is warranted for a smooth function.

1.3 Reviewer 1

Generally, point estimation provides poor information because it could vary with the simulations. For this reason, authors should provide estimated confidence intervals instead of just the single point estimation. Confidence intervals would give information regarding the robustness of the parameters' estimate together with an hint regarding the range in which the parameters should vary.

Answer:

The reviewer is correct. We therefore calculated the confidence intervals, according to the scheme presented in [<https://stat.ethz.ch/~stahel/courses/cheming/nlreg10E.pdf> ]. The text below, describing this, begins on line 334, in the Results section of the revised manuscript.

A 90% confidence interval for the parameter *p_c_* is obtained in the interval (0.978, 1.062) of its value, evaluated by the search. The sensitivity analysis further showed that parameter $k_{C}^{03}$ was the only parameter for which a distinct optimum is not obtained, since the system reaches saturation above the optimized value. Accordingly, the likelihood function receives a constant value for all values above the optimized value, and therefore the appropriate definition for a confidence interval in this case would be the range between the optimized value and the upper parameter range limit, which was determined as described in the Supporting Information.

1.4. Reviewer 1

It is not clear why the authors choose to vary the parameters in 1:63-fold range above and below the estimated baseline values, maybe this choice should be discussed a bit.

Answer:

The reviewer is correct, and the relevant text, starting on line 356 in the revised Manuscript

To examine the extent of change in cancer dynamics as a function of the change in the value of $k_{E}^{0}$ , the value for $k_{E}^{0}$ obtained for patient O’ was iteratively multiplied by 1.05 (representing a 5% increase between successive sampled values) ten times, and in addition, it was subsequently divided by 1.05 ten times, to thereby obtain a *1.05^10^=1.63*-fold range above and below the estimated baseline value for Patient O’.

1.5 Reviewer 1

Regarding the classification between responders and non-responders obtained in the 4 virtual populations, the plots in figure 4 do not show an evident clustering of the two groups but Table 1 gives a description of the obtained results. However, the method adopted to evaluate the classification accuracy is not given, therefore it would be worthwhile to add more details.

Answer:

The method for performing the classification and determining its accuracy is detailed in the original Supporting Information text. Hence, we inserted the following text in line 423 of the revised manuscript:

The method for performing the classification and determining its accuracy is detailed in the Supporting Information.

1.6 Reviewer 1

Please find attached all the comments and typos.

Answer

The appropriate corrections were made throughout the entire manuscript.

Reviewer #2: Comments (General):

2.1 Reviewer 2

The authors state, "Simulations of virtual populations were in good agreement with clinical results." It was not immediately apparent which clinical results are being used for comparison data. The first reading of this seems to imply that there will be a comparison to clinical trial population level data, but it is difficult to see where that is done. For the sake of validation, is there a clinical study against which the virtual population outcomes can be compared?

Answer:

Indeed, our work includes a comparison of model simulation results to results of a clinical trial, carried out to check the validity of a newly suggested response predictor, namely, the ratio between the initial tumor size and reinvigoration after beginning immunotherapy. The above statement in the reviewer’s comment (“Simulations of virtual…”) is from the abstract, where no references appear. However, in many places in the text (both in the Materials and Methods, the Results, the Discussion and the Supporting Information sections) there are clear references to Huang et al., Nature 2017, and to the relevant figure in their article, featuring the results of their clinical trial.

To avoid further confusion, we altered one sentence (L. 44) in the Abstract (Results) to read:

Simulations of a specific clinical trial were in good agreement with the clinical results, demonstrating positive correlations between response to pembrolizumab and the ratio of reinvigoration to baseline tumor load.

2.2 Reviewer 2

The approach of creating a within-host model and then simulating a population using that model is a good one, and has been implemented in other works. It would help the reader if the authors were to cite other works that have taken a similar approach, and clarify in what ways their approach is similar or differs. Individual to population simulation work has been done in, for example, http://www.sciencedomain.org/abstract/3987.

Answer:

We thank the reviewer for suggesting to expand on the concept of Virtual Clinical Trials. Accordingly, we added the following text, beginning on L. 124 in the revised manuscript and in the Discussion, L. 503:

The virtual, or *synthetic*, patient population approach, of creating a within-patient model and then simulating clinical trials in populations of such patients, was introduced by Agur and colleagues in early 2000s [1-4] and by Bangs [5]. According to this approach, a virtual physiology. A virtual patient-population is a collection of such virtual patients, each characterized by a set of parameters drawn from the distributions of these parameters in the real patient-population. Once a virtual patient population is established it can undergo virtual clinical trials, end points of which being those employed in the pharmaceutical industry, such as Progression-Free Survival (PFS), Objective Response Rate (ORR), etc. [3, 6]. The concepts underlying virtual clinical trials were employed for checking how prematurely shelved drugs can be rescued. Studying a discontinued drug, ISIS-5132, the Virtual Clinical Trial analysis showed that by combining the molecule in development, ISIS-5132, with a licensed drug, sunitinib malate (Sutent®, Pfizer Inc), the treatment of prostate cancer could be improved, with more patients reaching PFS at five years, as compared to either ISIS-5132 or sunitinib malate monotherapy [2].

In the present work, we employed these concepts to retrieve and analyze the clinical trial of [7], showing that in melanoma, the ratio of reinvigoration rate to baseline tumor load can be used to cluster patients according to the quality of their response. Our methodology involved the creation of plural virtual patient populations that differed in specific assumptions about the parametric relationships, and the use of these virtual populations to perform virtual trials which resemble those of Huang et al. This methodology was instrumental in deciphering the crucial intrinsic properties which underlie the reinvigoration/basic tumor load ratio in [7]. Taken together, our Virtual Clinical Trials simulating the “true” clinical trial in [7] further confirm the validity of our model, and demonstrate how different decisive personal parameters that are not necessarily estimable in the clinical setting, can be combined in a complex manner into one quantifiable measure, which can serve to classify responders.

2.3 Reviewer 2

The model may have potential for personalizing this MAB treatment approach, and further validation would be crucial. To that end, if readers can experiment with the model, testing and validation could happen more rapidly. There should be sufficient information provided to allow the model to be fairly easily implemented and simulation results reproduced by an interested reader. The authors state that the simulations were run in Matlab. It would be very helpful if the authors made the code both for the simulations and for the parameter fitting schemes available for testing purposes

Answer:

All simulation results are **completely** **reproducible** from the information already available in the document. New model simulations too are completely creatable using the current information in the document.

1. In the manuscript and in the Supporting Information Appendix S1 we present a full table of the parameters and the optimized values for Patient O’.
2. All the model’s equations (all variables and parameters included), are described to the full in the Supporting Information.
3. A description of the specific matlab solver used is also described in the Materials and Methods section.
4. The parameter fitting scheme is the ‘tailored algorithm’ that we describe in the revised version of the paper in the context of comment 1.1 of the 1^st^ reviewer.

As our algorithm is a sensible combination of several different algorithms, and marginally outperforms matlab’s built-in algorithms, it will be published in a separate paper, once tested on an independent modelling system*.*

Comments (Minor):

2.4 Reviewer 2

In Figure 1a, the authors state that "The cancer cells (khaki ellipses) divide by a power-law function, whose exponent is 2/3." A citation to the power-law growth model is provided later in line 205. The citation is a study done in breast cancer, yet the cancer being modeled in this paper is melanoma. Since it has been observed that different cancer types may grow according to different intrinsic growth laws, wt would help the reader if there were some further explanation as to why the power law, with exponent 2/3, was selected (as opposed to any other "popular" intrinsic growth law, such as Gompertzian or logistic).

Answer:

Indeed, in our original mid 1990’s work, the power growth law of solid tumors was extracted from large scale mammography screening trial data in breast cancer. However, our further work on several solid tumor indications confirmed the generality of the power law growth function for solid tumor under no medical intervention.

Accordingly, we replaced the original sentence (“The cancer cells etc..”) by the following two paragraphs, beginning on line 161 in the revised manuscript:

We assumed that in the absence of inhibition by immune cells, the tumor grows according to a power-law function, at a rate higher than linear yet lower than exponential (exponent equaling 2/3). This assumption was validated by *in vitro* and *in vivo* results in several cancer indications. In particular, large scale mammography screening trial data in breast cancer demonstrate power law growth (exponent approximating 1/2), but show inconsistency with both Gompertz and logistic growth functions [8]. Prospective validation of the power growth law was obtained by one-, two- and three-dimensional tumor growth experiments both in vitro, in MCF-7 cells (breast cancer cell line) and in vivo, in mouse xenografts. In all studied cases, the unsaturated growth follows a power law function with exponent 2/3 in the three-dimensional case [9]. In another work, the power law growth assumption was tested experimentally in implanted human ovarian carcinoma spheroids. Results show that initially similar tumor spheroids varied widely in their growth-rates. Ranging from almost exponential growth, to power law growth with different smaller exponents [10]. In a work on hormone sensitive prostate cancer we employed clinical measurements of Prostate-Specific Antigen (PSA) from a dataset of 83 ADT-treated HSPC patients, to show that a mechanistic model, including a tumor growth law with a dynamic power, successfully retrieved individual disease time-courses. In this work, simulations of other tumor growth functions showed a less accurate fit to the clinical patient data [11]. Taken together, these experimental and theoretical results, in different solid tumor indications and states, suggest that the power growth law is a general function describing intrinsic solid tumor growth.

2.5 Reviewer 2

Further clarification of the biological background would go a long way toward help a non-specialist audience appreciate this work. For example, it would help to further clarify the background provided on this treatment approach. Background is already provided by the authors, but the discussion could be clearer. An example of an improved explanation of what PD-1 checkpoints are and why checkpoint inhibitors are a treatment approach of interest can be found *here:*

<https://www.ncbi.nlm.nih.gov/pmc/articles/PMC6129935/>

Answer:

For further clarification of the mechanism of action of immune checkpoint inhibitors, we have replaced the third paragraph in the introduction, L. 80, by the following paragraph:

The effect of ICI is governed by complex interactive dynamics of the patient’s immune system, the growing tumor and the ICI drug. In pathogenic diseases in humans, T lymphocytes expand extensively upon encountering foreign antigens, and following the peak of expansion, the resolution of the inflammation and the clearance of the antigen, this expanded T cell population undergoes apoptosis. The mechanism underlying this immune regulation involves two main players: programmed cell death 1 (PD-1) - an immunoinhibitory receptor, expressed on T and B cells, and its ligand - programmed cell death ligand (PD-L) 1, expressed on T cells, B cells, macrophages, dendritic cells, and nonimmune cells. The expression of PD-L1 and the subsequent binding to PD-1 induces T cells to undergo apoptosis, thus containing the expansion of the immune response [14]. Melanoma cells can hijack this mechanism by expressing the same co-inhibitory signal, PD-L1, within the tumor microenvironment. These PD-L1 molecules bind to PD-1 receptors on T lymphocyte, stimulating these cells to undergo apoptosis, weakening the immune response prematurely, and hampering cancer cell clearance [12]. The ICI drugs were engineered to disrupt this cancer-induced ligand–receptor association and premature apoptosis. Pembrolizumab, for example, blocks the PD-L1,2 inhibitory pathway using anti-PD-1 antibodies. As a result, T cells can be re-activated, become more efficient in tumor surveillance, and enable the restoration of anticancer immunity and the suppression of cancer growth [13].

2.6 Reviewer 2

A straightforward definition of pembrolizumab up front would also help. For example, from <https://www.cancer.gov/publications/dictionaries/cancer-terms/def/pembrolizumab>: Pembrolizumab binds to a protein called PD-1, which is found on T cells. Pembrolizumab may block PD-1 and help the immune system kill cancer cells. It is a type of monoclonal antibody and a type of immune checkpoint inhibitor. Also called Keytruda.

Answer:

The reviewer is correct. Such definition has been added to line XXX of the newly described document (see answer to comment 2.5).

2.7 Reviewer 2

Figure 1a. Some elements of the figure are so closely overlapping that it makes the figure difficult to read.

Answer:

Notations were removed from Figure 1a, to present a clear and non-overloaded figure.

1.	Arakelyan L, Belilty G, Dahan N, Harpak H, Kogan Y, Merbl Y, et al. Application of the Virtual Cancer Patient Engine (VCPE) for improving oncological treatment desig. Journal of clinical oncology : official journal of the American Society of Clinical Oncology. 2004;V 22 (14 suppl):692.

2.	Kleiman M, Sagi Y, Bloch N, Agur Z. Use of virtual patient populations for rescuing discontinued drug candidates and for reducing the number of patients in clinical trials. Altern Lab Anim. 2009;37 Suppl 1:39-45. doi: 10.1177/026119290903701S07. PubMed PMID: 19807203.

3.	Agur Z. From the evolution of toxin resistance to virtual clinical trials: the role of mathematical models in oncology. Future Oncol. 2010;6(6):917-27. doi: 10.2217/fon.10.61. PubMed PMID: 20528230.

4.	Agur Z, Bloch N, Gorelik B, Kleiman M, Kogan Y, Sagi Y, et al. Developing Oncology Drugs Using Virtual Patients of Vascular Tumor Diseases. 2011. p. 201-37.

5.	Bangs A. Predictive biosimulation and virtual patients in pharmaceutical R&D. Studies in health technology and informatics. 2005;111:37-42.

6.	Agur Z. Interactive Clinical Trial Design ” : A Combined Mathematical and Statistical Simulation Method for Optimizing Drug Development from Statistical Methods in Healthcare. Faltin FW, Kenett R, F. R, editors: Wiley; 2012.

7.	Huang AC, Postow MA, Orlowski RJ, Mick R, Bengsch B, Manne S, et al. T-cell invigoration to tumour burden ratio associated with anti-PD-1 response. Nature. 2017;545(7652):60-5. doi: 10.1038/nature22079. PubMed PMID: 28397821; PubMed Central PMCID: PMC5554367.

8.	Hart D, Shochat E, Agur Z. The growth law of primary breast cancer as inferred from mammography screening trials data. Br J Cancer. 1998;78(3):382-7. PubMed PMID: 9703287; PubMed Central PMCID: PMC2063020.

9.	Hochman G, Agur Z. Deciphering Fate Decision in Normal and Cancer Stem Cells: Mathematical Models and Their Experimental Verification in Mathematical Methods and Models in Biomedicine: Springer; 2013.

10.	Arakelyan L, Merbl Y, Agur Z. Vessel maturation effects on tumour growth: validation of a computer model in implanted human ovarian carcinoma spheroids. Eur J Cancer. 2005;41(1):159-67. doi: 10.1016/j.ejca.2004.09.012. PubMed PMID: 15618001.

11.	Elishmereni M, Kheifetz Y, Shukrun I, Bevan GH, Nandy D, McKenzie KM, et al. Predicting time to castration resistance in hormone sensitive prostate cancer by a personalization algorithm based on a mechanistic model integrating patient data. Prostate. 2016;76(1):48-57. doi: 10.1002/pros.23099. PubMed PMID: 26419619.

12.	Trivedi MS, Hoffner B, Winkelmann JL, Abbott ME, Hamid O, Carvajal RD. Programmed death 1 immune checkpoint inhibitors. Clin Adv Hematol Oncol. 2015;13(12):858-68. PubMed PMID: 27058852.

13.	Sharma P, Allison JP. The future of immune checkpoint therapy. Science. 2015;348(6230):56-61. doi: 10.1126/science.aaa8172. PubMed PMID: 25838373.
